# The Components of Self-Perceived Health in the Kailali District of Nepal: A Cross-Sectional Survey

**DOI:** 10.3390/ijerph120303215

**Published:** 2015-03-17

**Authors:** Leila Freidoony, Ranabhat Chhabi, Chang Soo Kim, Myung Bae Park, Chun-Bae Kim

**Affiliations:** 1 Institute for Poverty Alleviation and International Development, Yonsei University, 1 Yonseidae-Gil, Gangwon-Do, Wonju City 220-710, Korea; E-Mails: l.freidoony@yonsei.ac.kr (L.F.); chhabir@gmail.com (R.C.); kimc@yonsei.ac.kr (C.S.K.); parklove5004@naver.com (M.B.P.); 2 Department of Preventive Medicine, Wonju College of Medicine, Yonsei University, 20 Ilsan-Ro, Gangwon-Do, Wonju City 220-701, Korea; 3 Department of Business Administration, College of Government and Business, Yonsei University, 1 Yonseidae-Gil, Gangwon-Do, Wonju City 220-710, Korea; 4 Department of Health Administration, Yonsei University, 1 Yonseidae-Gil, Gangwon-Do, Wonju City 220-710, Korea; 5 Healthy City Research Center, Institute of Health and Welfare, Yonsei University, 1 Yonseidae-Gil, Gangwon-Do, Wonju City 220-710, Korea

**Keywords:** self-perceived health, health behaviors, happiness level, satisfaction with healthcare services, cross-sectional, Nepal

## Abstract

Self-perceived health is a health measure with well-established links with mortality, healthcare services utilization, and future health. Various components of self-perceived health have been identified in different populations. In this study, we aimed to investigate the components of self-perceived health in a Nepali population. This was a cross-sectional survey conducted in the Kailali district of Nepal in 2014. The sample was initially consisted of 309 households, representative of the population of one municipality and one village; however, 304 participants were included in the analyses. Information on socio-demographic characteristics, health condition, satisfaction with healthcare services, psychological factors, and health behaviors was extracted. Logistic regression analyses were carried out to identify putative components of self-perceived health. Among the 304 respondents, 244 (80.3%) and 60 (19.7%) perceived their health as good and poor, respectively. Middle age and lower satisfaction with healthcare services were associated with worse self-perceived health, accounting for 10.3% of variance. No regular exercise, drinking, smoking, and being unhappy were also related with worse self-perceived health, after adjustment for age and satisfaction level. In the final model, however, drinking status did not significantly contribute. Our findings support previous findings that individuals with positive health behaviors and psychological wellbeing are more likely to perceive their health better. This study may direct public health policies toward more targeted interventions.

## 1. Introduction

Self-perceived health (also known as subjective overall health or self-rated health) is one of the most commonly used health measures in surveys. This measure reflects respondents’ overall perceptions of their general health status. It is typically obtained by a simple question such as “How is your health status?” or “How do you perceive your health situation?” on a four or five-point scale. Since self-perceived health provides information that cannot be reached by other health measurements, it is regarded as an inclusive and popular measure in health surveys and clinical studies [1].

Previous studies have consistently reported that self-perceived health is one of the best predictors of future health, use of healthcare services and corresponding costs, mortality patterns, recovery from illness, and quality of life [2,3,4,5,6,7]. Regarding general practice, self-perceived health can be a good screening tool for general health assessment, especially in poor countries where medical facilities for examining risk factors such as high cholesterol, hypertension, diabetes, *etc.* are not widely available and accessible. In such situations, self-perceived health may be a surrogate method to assess the health status of a population because of its simplicity and its well-established links with the aforementioned health indicators [6].

Self-perceived health has different predictors across populations. Identifying the main components of self-perceived health in a specific context has several public health benefits such as the ability to better address cultural and specific characteristics of the population in public health policies and to implement more targeted interventions for that population. Several socio-demographic, socioeconomic, health conditions, health behaviors, and psychosocial factors have been shown to be predictors of self-perceived health in different populations [8,9,10,11,12,13,14]. In general, women, individuals with low socioeconomic status and older people tend to perceive their health more negatively. However, controversy remains, especially over the impact of gender [15,16,17]. Moreover, health conditions (such as the presence of chronic diseases and physical symptoms) constitute the principal components of self-perceived health [10,18]. Results of research on health behaviors and psychological factors vary across populations, but they show a common pattern of better self-perceived health by those who adopt positive health behaviors and are in a good state psychologically [8,11,19,20,21].

Several studies conducted in developed (high/middle-income) countries have examined the multi-dimensional nature of self-perceived health and its components [22,23,24]; the main focus in the recent researches is on determining any relevant disparity, and on modeling the identified associations. However, very few studies have been conducted using data from underdeveloped countries (low-income countries) [10,25], largely because little information is available on the potential key components of self-perceived health in those countries. In addition, these studies are still in their infancy, mostly have cross-sectional design, and the main focus is on determining the corresponding components in the general population. Information on self-perceived health in the context of Nepal is very incomplete and in the early stages of development. Existing studies have focused on a specific population such as internally displaced persons or older adults, using socioeconomic status, disease-related, and psychosocial factors as determinants of self-perceived health [26,27].

Although the Nepalese healthcare system and basic health services has been improved and developed during the last decade, there are still many challenges such as lack of infrastructure, financial resources, equipment, supplies, trained staff, electricity, transportation and water supplies. On the other hand, Nepal is one of the least-developed countries in the World and one fourth of its population lives below the national poverty line [28]; so, it is important to better understand the main components of self-perceived health as a low-cost surrogate measure of health status. The aim of this study is to investigate the influential factors of self-perceived health, with particular emphasis on health behaviors and psychological factors.

## 2. Experimental Section

### 2.1. Study Area and Design

This study was based on data from the 2014 Baseline Survey in the Kailali district of Nepal conducted by the Institute for Poverty Alleviation and International Development (IPAID). IPAID is a university-based research institution which has taken a multi-disciplinary approach to policy research on global poverty alleviation, conducting several baseline and follow up surveys in poor and under-developed countries such as Nepal.

The 2014 Nepal Baseline Survey was part of a large ongoing cooperation between Yonsei University and Good Neighbors International in the Kailali district. The data were collected between February and March 2014 by semi-structured interview of a sample of households residing in the Tikapur municipality and the Narayanpur Village Development Committee (VDC). The study areas were selected purposively. It sought to assess the baseline agriculture, economy, health, and social network status of individuals in the aforementioned areas.

A cross-sectional design was selected for this study because we sought to find out the associated components of self-perceived health in a rather short time to provide a better insight into the issue for policy makers, and to generate hypotheses on the topic for further research [29].

This cross-sectional survey was initially based on a sample of 309 households drawn randomly using cluster sampling to include various geographical regions. The following formula was used for sample selection:

$$\text{Sample}\;\text{size}=\frac{{\text{Z}}^{2}\times \text{p}\times \left(1-\text{p}\right)}{{\text{c}}^{2}}$$

where, Z = Z value (1.96 for 95% confidence level); p = percentage picking a choice (0.5); c = confidence interval or absolute precision (5.6%).

In principle, no eligibility criteria were used and one member of each household (mainly head of household) was surveyed unless he/she refused. Altogether, 305 households (mean age 40.3 years) were surveyed out of 309 sampled, for a response rate of 98.7%.

### 2.2. Data Instrument and Collection

The baseline survey was conducted between February and March 2014 through face-to-face interviews in each household sampled from Tikapur municipality and Narayanpur VDC by trained IPAID surveyors. A multi-item questionnaire was developed based on a review of the relevant literature and subsequently translated into Nepali language. The questionnaire collected information on agriculture, economic, health, and social network status of the sample. A pilot study was conducted and the questionnaire was pre-tested in a nearby VDC with similar context.

Data collection was completed by trained field researchers who were familiar with research areas and culture. They received intensive training to get the adequate information and to minimize the errors. The surveyors also were provided with interview guidelines.

### 2.3. Data Management and Ethical Consideration

The health questionnaire consisted of several sections addressing various health topics: descriptions of health status, disability and functional limitations, healthy living, health behaviors, and satisfaction with healthcare services. In this analysis, we considered questions related to healthy living, health behaviors, and satisfaction level. Socio-demographic data including age, gender, marital status, number of family members, and total income were obtained from the IPAID composition survey.

In total, 305 individuals 12 years or older answered the questionnaires. Of those, one observation (0.3%) was excluded from the present analysis due to missing data on self-perceived health (dependent variable). Values for missing data (84, 2.13% missing values) on all the independent variables, except for educational level, were treated by multiple imputations using chained equations [30] to deal with the problem of missing observations in the multivariable analyses. Multiple imputation by chained equations (MICE), sometimes called “fully conditional specification” or “sequential regression multiple imputation” is a multiple imputation method well described in IBM Corp. (Chicago, IL, USA) [31]. In short, MICE iteratively complete missing values in different variables by using chained equations, which are univariate imputation models. Then, fully conditional specification is used for the prediction equations. All variables, except the one to be imputed, were included in the prediction equation. By default, five different sets of data are imputed to consider the uncertainty around the missing values. Descriptive characteristics of the sample prior to imputation of the missing data have been represented in Table S1.

All subjects gave their informed consent [32,33] for inclusion before they participated in the study. The study was conducted in accordance with the Declaration of Helsinki, and the protocol was approved by the Ethics Committee of Yonsei University (NRF-2013S1A5B8A01055336) and local government of Kailali district, Nepal.

### 2.4. Measurements

#### 2.4.1. Dependent Variable

Self-perceived health was evaluated with the following question: “How is your health status? How do you perceive your health situation”? Response options were on a six-point scale including: 1 = positive (excellent) health, 2 = better (good) health, 3 = relief from illness, 4 = unrecognized illness, 5 = mild illness, and 6 = severe illness. For the main analyses, responses were recoded using a two-point scale: 1 = “Poor” category included “severe illnesses”, “mild illnesses”, “unrecognized illnesses”, and “relief from illness”; and 2 = “Good” category included “better health” and “positive health”. “Good” self-perceived health served as the reference category in the analyses.

#### 2.4.2. Independent Variables

We also examined socio-demographic factors (gender, age, marital status, household size, and total income), health behaviors (smoking, alcohol consumption, and regular exercise), health condition (history of any chronic disease), psychological factors (happiness level and any suicide attempt), and satisfaction level with healthcare services.

Socio-demographic variables retained for this analysis included gender, age at the time of survey (grouped into three categories: <45, 45–65, >65 years old [19]), marital status of head of household, household size, and total household income. Income was measured as monthly net income from all sources. Income quintiles were subsequently constructed and further adjusted for the household size (using square root scale [34]). The “education level” variable (with 34.1% missing data proportion) was excluded from the analysis due to some reasons: (1) coding error for “no education” and “no response”; subsequently, the number of respondents who were actually illiterate may have been far underestimated; (2) results of the Little’s Missing Completely at Random (MCAR) Test [35] when education level was included, showed the pattern of Missing Not at Random (MNAR). This implies that multiple imputation is not a suitable method for the data imputation; (3) education data of corresponding respondents was extracted indirectly from the Composition Survey (done along with the Baseline Survey), which inevitably decreases the reliability of the data.

Health behavior variables included smoking, drinking, and regular exercise. Regarding smoking status, participants were classified as “current smokers” if they reported smoking currently, “former smokers” if they had quit smoking, and “never” if they had never smoked. A similar classification was also applied for drinking status. Exercise was measured by asking, “Do you regularly exercise?” with two responses of “Yes” or “No”.

Chronic diseases, as an indicator of health condition, were measured by asking respondents to indicate whether they had ever suffered from one of these conditions: diabetes mellitus, cancer, chronic obstructive pulmonary disease, or cardiovascular diseases. Answers were coded into two categories of “Yes” or “No”.

Psychological variables included happiness level and any suicide attempt. Happiness level was measured by asking “Have you been happy for the past week?” and answers were classified as “happy”, “moderate”, and “unhappy”. Respondents were also asked whether they had ever attempted suicide in the last year, with two responses of “Yes” or “No”.

The satisfaction variable included satisfaction with healthcare services. Using a six-point scale (1 = very dissatisfied; 2 = dissatisfied; 3 = somewhat dissatisfied; 4 = somewhat satisfied; 5 = satisfied; 6 = very satisfied), this questionnaire consisted of one question regarding satisfaction level with healthcare services: “How satisfied are you with healthcare services in your village”? For the analyses, answers were recorded into three categories: 1 = dissatisfied (including “very dissatisfied” and “dissatisfied”); 2 = fair (including “somewhat dissatisfied” and “somewhat satisfied”); and 3 = satisfied (including “very satisfied” and “satisfied”).

### 2.5. Statistical Analyses

First, bivariate associations of independent variables categories with self-perceived health categories were evaluated using chi-squared test and correlation analyses. Table 1 shows descriptive statistics (number and percentage values) and distribution of independent variables across two categories of self-perceived health, with *p* values of chi-squared tests and Kendall’s tau-b coefficients assisting the interpretation of direction of relationships.

Next, binary logistic regression models were used to examine the associations between self-perceived health and independent variables. We estimated four types of binary logistic regression models: (1) “unadjusted model” which evaluated the effect of each of the adjustment variables (including socio-demographic characteristics, health condition, and satisfaction level) separately; (2) “adjusted model (I)” for the aforementioned adjustment variables in which all of them were simultaneously included in the same model; (3) “adjusted model (II)” for the health behaviors and psychological factors in which we added each of these factors to the adjusted model (I); (4) “final model” which we simultaneously included all of independent variables in the same model to assess the net effect of each variable.

For the “unadjusted model”, socio-demographic, health condition, and satisfaction level variables (significant at *p* value < 0.1 in bivariate analyses) were first individually entered into binary logistic regression with self-perceived health as a dependent variable. For the “adjusted model (I)”, all of the aforementioned variables were simultaneously included in the same model. For the “adjusted model (II)”, each health behavior and psychological measure (significant at *p* value < 0.1 in bivariate analyses) was adjusted for the effects of the second model. Eventually, all independent variables were included in the “final model” simultaneously.

Statistical analyses were performed using IBM SPSS Statistics for Windows version 20.0 (IBM Corp., Armonk, NY, USA) [36]. In all analyses, pooled data from five iterations of multiple imputations were used. Unadjusted and adjusted odds ratios (OR), 95% confidence intervals (95% CI), and Nagelkerke R^2^ (which represents the amount of variability of self-perceived health explained by the models) are presented in Table 2, Table 3, and Table 4. The *p* level of significance was set at 0.05 for all analyses.

**Table 1:** Bivariate analyses of independent variables with self-perceived health categories (n = 304).

Variable	Good (n_1_ = 244)	Poor (n_2_ = 60)	*p* Value (Kendall’s Tau b ^a^)	Variable	Good (n_1_ = 244)	Poor (n_2_ = 60)	*p* Value (Kendall’s Tau b)
**Socio-demographic characteristics**				**Health behaviors**			
**Gender**			0.788 (0.015)	**Smoking**			0.006 ** (−0.181)
Male (%)	114 (79.7)	29 (20.3)		Current	50 (68.5)	23 (31.5)	
Female (%)	130 (80.7)	31 (19.3)		Former	9 (69.2)	4 (30.8)	
**Age groups**			0.093 **^†^** (0.110)	Never	185 (84.9)	33 (15.1)	
<45 (%)	169 (83.7)	33 (16.3)		**Drinking**			0.013 * (−0.142)
45–65 (%)	63 (72.4)	24 (27.6)		Current	65 (70.7)	27 (29.3)	
>65 (%)	12 (80.0)	3 (20.0)		Former	12 (85.7)	2 (14.3)	
**Marital status**			0.550 (−0.034)	Never	167 (84.3)	31 (15.7)	
Married	222 (79.9)	56 (20.1)		**Regular exercise**			0.011 * (0.146)
Other	22 (84.6)	4 (15.4)		Yes	32 (97.0)	1 (3.0)	
**Household size** (Mean)	6.40	5.92	0.235 (−0.068)	No	212 (78.2)	59 (21.8)	
**Income quintiles**			0.950 (0.028)	**Psychological factors**			
Q1 (lowest)	46 (76.7)	14 (23.3)		**Happiness level**			0.005 ** (0.148)
Q2	49 (80.3)	12 (19.7)		Happy	113 (85.6)	19 (14.4)	
Q3	50 (82.0)	11 (18.0)		Moderate	122 (78.7)	33 (21.3)	
Q4	50 (82.0)	11 (18.0)		Unhappy	9 (52.9)	8 (47.1)	
Q5 (highest)	49 (80.3)	12 (19.7)					
**Satisfaction status**				**Suicide attempt**			0.255 (−0.068)
**Healthcare services**			0.003 ** (−0.175)	Yes	4 (57.1)	3 (42.9)	
				No	240 (80.8)	57 (19.2)	
Dissatisfied	20 (72.1)	7 (27.9)		**Health condition**			
Fair	140 (75.3)	46 (24.7)		**Chronic disease**			0.062 **^†^** (−0.098)
Satisfied	84 (92.3)	7 (7.7)		Yes	16 (66.7)	8 (33.3)	
				No	228 (81.4)	52 (18.6)	

**^a^** Kendall’s tau b coefficient is a statistic used to measure the association between two quantities. In this table, positive values indicate poor perception as we vertically advance to independent variables’ categories and vice versa for the negative values. **^†^** Level of significance < 0.1; * Level of significance < 0.05; ** Level of significance < 0.01.

**Table 2:** Logistic regression analyses of the adjustment variables with self-perceived health (n = 304).

Variable	Unadjusted OR (95% CI)	Adjusted (Model I) OR ^1^ (95% CI)
**Age**		
<45 **^a^**	−	−
45–65	1.95 (1.07–3.55) *	1.87 (1.01–3.05) *
>65	1.25 (0.34–4.69)	1.37 (0.33–5.72)
**Chronic disease**		
Yes	2.32 (1.94–5.72) *	2.35 (0.91–6.07)
No **^a^**	−	−
**Satisfaction with healthcare services**		
Dissatisfied	4.12 (1.30–13.07) *	3.87 (1.21–12.44) *
Fair	3.95 (1.70–9.18) **	3.87 (1.65–9.09) **
Satisfied **^a^**	−	−

**^1^** Nagelkerke R^2^ = 10.3%, means that 10.3% of self-perceived health variance is explained by age, health condition and satisfaction level. **^a^** Reference group; OR: Odds Ratio; CI: Confidence interval. * Level of significance < 0.05; ** Level of significance < 0.01.

**Table 3:** Logistic regression analyses of health behavior and psychological factors with self-perceived health (n = 304).

Variable	Adjusted (Model II) OR ^1^ (95% CI)	Nagelkerke R^2^ Change ^2^
	Poor *vs.* Good	
**Smoking**		+4%
Current *vs.* Never **^a^**	2.57 (1.36–4.85) **	
Former *vs.* Never	1.72 (0.42–7.07)	
**Drinking**		+4.6%
Current *vs.* Never **^a^**	2.28 (1.21–4.30) *	
Former *vs.* Never	0.348 (0.04–3.01)	
**Regular exercise**		+4.1%
No *vs.* Yes **^a^**	4.92 (1.16–17.71) *	
**Happiness level**
Moderate *vs.* Happy **^a^**	1.62 (0.85–3.10)	+2.9%
Unhappy *vs.* Happy	3.88 (1.23–12.25) *	

**^1^** Adjusted for age, health condition, and satisfaction level; **^2^** Nagelkerke R^2^ change above the 10.3% of the first adjusted model (I) (see Table 2); **^a^** Reference group; OR: Odds Ratio; CI: Confidence interval; * Level of significance < 0.05; ** Level of significance < 0.01.

**Table 4:** Logistic regression analyses of all independent variables with self-perceived health (n = 304).

Variable	Final Model OR (95% CI) ^1^
**Age**	
45–65 *vs.* <45	1.95 (1.01–3.76) *
>65 *vs.* <45	1.79 (0.42–7.75)
**Chronic disease**	
Yes *vs.* No	2.10 (0.68–6.48)
**Satisfaction with healthcare services**	
Dissatisfied *vs.* Satisfied	3.66 (1.50–8.91) *
Fair *vs.* Satisfied	3.29 (0.96–11.26)
**Smoking**	
Current *vs.* Never	2.19 (1.03–4.05) *
Former *vs.* Never	1.98 (0.45–8.64)
**Drinking**	
Current *vs.* Never	1.60 (0.78–3.28)
Former *vs.* Never	0.23 (0.02–2.33)
**Regular exercise**	
No *vs.* Yes	4.36 (1.06–15.30) *
**Happiness level**	
Moderate *vs.* Happy	1.62 (0.83–3.15)
Unhappy *vs.* Happy	3.58 (1.06–12.08) *

**^1^** Nagelkerke R^2^ = 22.2%, means that 22.2% of self-perceived health variance is explained by the final model. OR: Odds Ratio; CI: Confidence Interval; * Level of significance < 0.05.

## 3. Results

### 3.1. Bivariate Analyses

Among the 304 participants, 244 (80.3%) and 60 (19.7%), perceived their health as good and poor, respectively. Table 1 presents the distribution of all independent variables across the categories of self-perceived health. The chi-squared tests, correlation analyses and Kendall’s tau *b* coefficients showed that all socio-demographic variables, suicide attempt, and health condition (any chronic disease) were not significantly associated with self-perceived health (*p* value > 0.05); however, this association was significant for age and health condition at *p* value < 0.1. On the other hand, and as might be expected, all health behavior variables, happiness level, and satisfaction level with healthcare services were significantly associated with self-perceived health (*p* < 0.05). Non-smokers, non-drinkers, and those performing regular exercise perceived themselves as healthier. Happier individuals and those more satisfied with healthcare services also tended to perceive their health better than respondents who reported being unhappy and dissatisfied.

### 3.2. “Unadjusted” Model and “Adjusted” Model (I) for the Adjustment Variables

Table 2 presents the results of binary logistic regression models for self-perceived health. Crude and adjusted ORs for the main adjustment variables (significant at *p* value < 0.1 in the previous bivariate analyses) represent the likelihood of an individual to perceive his/her health as poor. In the first model, ORs greater than 1 for middle-aged individuals (45–65 years) and those who were not satisfied with healthcare services in their town/village indicated that these groups are more likely to perceive their health status as poor than younger or older individuals and those with complete satisfaction with healthcare services. Additionally, the unadjusted OR for poor *vs.* good self-perceived health revealed no significant difference between individuals >65 years *vs.* <45 years (OR 1.25, 95% CI 0.34–4.69). In brief, almost all unadjusted variables were significant (*p* < 0.05) in the first model with trends as described above. However, adjusted ORs in the adjusted model (I) decreased for all variables, with age and satisfaction level still remaining significant. In the “adjusted model (I)”, age, chronic disease, and satisfaction with healthcare services accounted for 10.3% of self-perceived health variance.

### 3.3. “Adjusted” Model (II) for Health Behavior and Psychological Variables

Table 3 presents the contribution of each health behavior and psychological variable to the self-perceived health, after adjusting for age, health condition, and satisfaction with healthcare services. Smoking, drinking, no regular exercise and unhappiness increased the likelihood of perceiving one’s health status as poor. Considering the Nagelkerke R^2^ increases, the contribution of health behavior and psychological factors to the self-perceived health was as follows: smoking 4.0%, drinking 4.6%, regular exercise 4.1%, and happiness level 2.9%. However, the category “Former” for smoking and drinking did not reach significance after adjustment for age, health condition, and satisfaction level variables. Moderate level of happiness also did not differ between those with poor *vs.* good self-perceived health. On the other hand, current smoking and drinking, no regular exercise and being unhappy were all significantly associated with poor self-perceived health.

In the “final model”, where all health behavior and psychological variables were simultaneously adjusted for age, health condition, and satisfaction level, corresponding ORs for all main variables were lower than those in previous models; however, age (OR = 1.95, 95% CI = 1.01–3.76 for the middle-aged group), satisfaction level with healthcare services (OR = 3.66, 95% CI = 1.50–8.91 for dissatisfied respondents), smoking status (OR = 2.19, 95% CI = 1.03–4.05 for current smokers), regular exercise (OR = 4.36, 95% CI = 1.06–15.30 for those not performing), and happiness level (OR = 3.58, 95% CI = 1.06–12.08 for unhappy individuals) all remained significant predictors of self-perceived health. On the other hand, the significance level for drinking status and fair satisfaction declined, so that these factors were no longer significantly associated with self-perceived health. The significance level of smoking status also declined considerably.

## 4. Discussion

In this cross-sectional study, we sought to investigate the components of self-perceived health in a sample population from the Kailali district of Nepal, with an emphasis on health behaviors and psychological factors. We found strong associations between self-perceived health and a number of health behaviors and psychological factors; regular exercise and happiness level showed the strongest associations with self-perceived health.

Age, health condition and level of satisfaction with healthcare services accounted for 10.3% of variation in self-perceived health. Adjusted ORs showed that age and satisfaction with healthcare services are more potent determinants of self-perceived health than health condition. Regarding health behaviors and psychological factors, no regular exercise, current smoking, and being unhappy were all related to worse self-perceived health. Additionally, the relationship between self-perceived health and drinking status was shown to be non-significant after controlling for the other factors in the final model. These results suggest that, in the context of this study, self-perceived health is more greatly affected by health behaviors and psychological factors than socio-demographic factors.

In spite of findings from several studies in different contexts [14,15], the distributions of almost all socio-demographic variables across the two groups of self-perceived health in our sample were not significantly different, so they were not included in the next analyses. The small sample size and different culture and context might be an explanation for this variation; cultural patterns may affect the responses. In addition, different physical symptoms and psychosocial factors may cause different populations to perceive their health less or more positively. However, our findings were consistent with other studies with regard to age categories [10,19].

Various studies have shown that the number of older people who perceive themselves as healthy decreases with age, and therefore aging is highly associated with worse self-perceived health [16,37]. However, our results highlight the middle-aged disadvantage in self-perceived health and this disadvantage persisted even after adjusting for other variables. On the other hand, the elderly perceived their health better than the middle-aged. This discrepancy may be due to physical symptoms and psychosocial factors which differently affect age groups. According to the social comparison theory, the elderly have lower expectations regarding health than do the young [6], and these expectations may result in more positive self-perceived health among the elderly and more negative self-perceived health in the young [38]. Additionally, young and middle-aged groups are the main workforce in Nepal [39], and these groups face work hazards and stresses.

Impaired health condition (in this study assessed as suffering from any chronic disease) is a well-established component of poor self-perceived health [18,40]. In our study, though significant in the unadjusted model, the addition of age and satisfaction variables removed the effects of health condition, suggesting that age and satisfaction level may act as confounders for health condition. Additionally, this may be due to the limited number of chronic disease conditions presented to respondents; therefore, frequency of suffering from chronic diseases might be underestimated in our study. On the other hand, chronic diseases such as diabetes, either in the early stages or well-controlled, do not impair individuals’ functionality and quality of life, and consequently do not affect individuals’ health perception [41].

Respondents dissatisfied with healthcare services were more likely to report their health as poor. Satisfaction can be defined as the distance between an individual’s experience and his or her expectations. Patient satisfaction is associated with the sense of fulfillment of their general and condition-specific health care needs. Unmet needs lead to chronic diseases, which ultimately render individuals less positive about their health status [42].

Health behaviors and psychological factors are well-known components of better self-perceived health [11,21]. Our study also confirms these findings, although the measurements were not based on standard questionnaires. Regular exercise, no smoking, and higher level of happiness were associated with better self-perceived health. Drinking, though significant in the adjusted model (II), was not a significant contributor to self-perceived health after controlling for other factors. This finding is inconsistent with previous studies and may be due to the theory that individuals may not perceive their health as poor unless they can establish a direct connection between their behaviors and functional limitations in their mind [43]. As might be expected, regular exercise is an important contributor to self-perceived health among different age groups and in different contexts [21,44,45]. Furthermore, performing regular exercise is associated with positive effects on mood and wellbeing [46], and psychological wellbeing is in turn related to better self-perceived health [40]. Smoking has been also documented to be significantly associated with poor self-perceived health [21,47].

Concerning psychological factors, as previously mentioned, we first included two variables: suicide attempt and happiness level. Both have been reported to be important predictors of self-perceived health [20,48]. However, suicide was then excluded from the analyses due to insignificant distribution in the chi-squared test. Regarding happiness level, Siahpush and colleagues [20] showed that happier people are more likely to prospectively perceive their health more positively.

On the whole, our final model with the aforementioned variables partly explained the variance of self-perceived health in the study sample. This demonstrates that several factors should be included in a model to explain and predict self-perceived health in different settings and samples. Among these factors, psychosocial variables such as stress should be of special interest. Psychosocial contributors, directly or indirectly related to stress, seem very promising as maladaptive responses to stress may lead to a broad range of behavioral and physical changes and ultimately, negative health behavior patterns and physical exhaustion [49]. Other contributing factors would be socioeconomic status, measures of physical and mental health, social relationships and networks, environmental perceptions, spirituality, and other health behavior variables such as quality of sleep and eating patterns.

### Strengths and Limitations

The strength of the present study lies in the fact that there is scarce data on self-perceived health status in the general population of Nepal. Our findings may shed more light on the multidimensional nature of self-perceived health in this poor country.

However, this study has certain limitations that should be considered. First, the sample size was small with a rather wide confidence interval, meaning that the results are non-generalizable and slightly imprecise. Second, the cross-sectional design limits any inference about the direction of relationships among variables or causality inference. Third, the measure of health condition (implied by presence of any chronic disease) and most of the other measures were self-reported, subjecting them to recall bias and underestimation of their effects on self-perceived health. Fourth, we used simple and non-standard questions to assess health behaviors and psychological factors which are essentially complex factors; hence, the validity of our measurements may have been weakened. More precise questions including additional factors should be considered for future studies. Fifth, notwithstanding the diverse problematic issues [50,51,52], we inevitably had to categorize the dependent variable as well as the independent variables for the binary logistic regression models. Finally, these findings reflect the participants’ current situations. Longitudinal studies are needed to track changes over time.

## 5. Conclusions

Until now, few studies have been conducted in Nepal to investigate the main components of self-perceived health, especially taking into consideration health behaviors and psychological factors. In this respect, the present study makes a significant contribution because it shows that health behaviors and psychological factors are important and potential elements that contribute to self-perceived health. Since there is a close association between self-perceived health and future morbidity and mortality, strengthening it is an important issue for public health policies. Therefore, in the study context, holistic approaches should be targeted at health promoting behaviors and psychological aspects of individuals’ lives, along with improving healthcare services. In this regard, governmental policies and mass media campaigns may play a key role. However, in-depth and longitudinal studies with larger sample sizes and more factors are also needed to precisely assess the components of self-perceived health in the Nepali population and to implement interventions more efficiently.

## Supplementary Files

Supplementary File 1
