# Supplementary material for: The Components of Self-Perceived Health in the Kailali District of Nepal: A Cross-Sectional Survey
Source: Int J Environ Res Public Health. 2015 Mar 17;12(3):3215–31. doi: 10.3390/ijerph120303215 (PMC4377960; doi:10.3390/ijerph120303215)
Supplement: Supplementary File 1 [file ijerph-12-03215-s001.pdf]

The Components of Self-Perceived Health in the Kailali District of Nepal: A Cross-Sectional Survey

Table S1. Descriptive characteristics of the sample *.

**Characteristics**	**No. or Range**	**% or Mean (SD)**
Gender *(Respondents: 302)*		
Male	160	52.98
Female	142	47.02
Age in years *(Respondents: 302)*	12–84	40.32 (14.72)
Marital Status *(Respondents: 300)*		
Married	274	91.33
Other	26	8.67
Family size *(Respondents:304)*	2–20	6.31 (2.50)
Education level *(Respondents: 200)*		
Primary	85	42.50
Lower Secondary	40	20.00
Secondary and Upper Secondary	63	31.50
University	12	2.00
Income in Nepalese Rupee *(Respondents: 263)*	2000–1,208,000	106,283.81 (171,248.11)
Self-perceived Health *(Respondents: 304)*		
Good	244	80.30
Poor	60	19.70
Drinking *(Respondents: 289)*		
Current	89	30.80
Former	12	4.15
Never	188	65.05
Smoking *(Respondents: 300)*		
Current	73	24.33
Former	12	4.00
Never	215	71.67
Regular Exercise *(Respondents: 299)*		
Yes	32	10.70
No	267	89.30
Happiness Level *(Respondents: 302)*		
Happy	131	43.38
Moderate	154	50.99
Unhappy	17	5.63
Suicide attempt *(Respondents: 300)*		
Yes	6	2.00
No	294	98.00
Chronic disease *(Respondents: 301)*		
Yes	24	7.97
No	277	92.03

Table S1. *Cont.*

**Characteristics**	**No. or Range**	**% or Mean (SD)**
Satisfaction with healthcare services *(Respondents: 299)*		
Dissatisfied	27	9.03
Fair	183	61.20
Satisfied	79	26.42

* Prior to imputation of the missing data.

© 2015 by the authors; licensee MDPI, Basel, Switzerland. This article is an open access article distributed under the terms and conditions of the Creative Commons Attribution license (http://creativecommons.org/licenses/by/4.0/).
